# Supplementary material for: Wnt signaling promotes inflammation and EMT-associated gene expression in mesenchymal TNBC
Source: Sci Rep. 2026 Apr 2;16:15798. doi: 10.1038/s41598-026-43678-3 (PMC13194965; doi:10.1038/s41598-026-43678-3)
Supplement: Supplementary file 1 — Supplementary Material 1 [file 41598_2026_43678_MOESM1_ESM.docx]

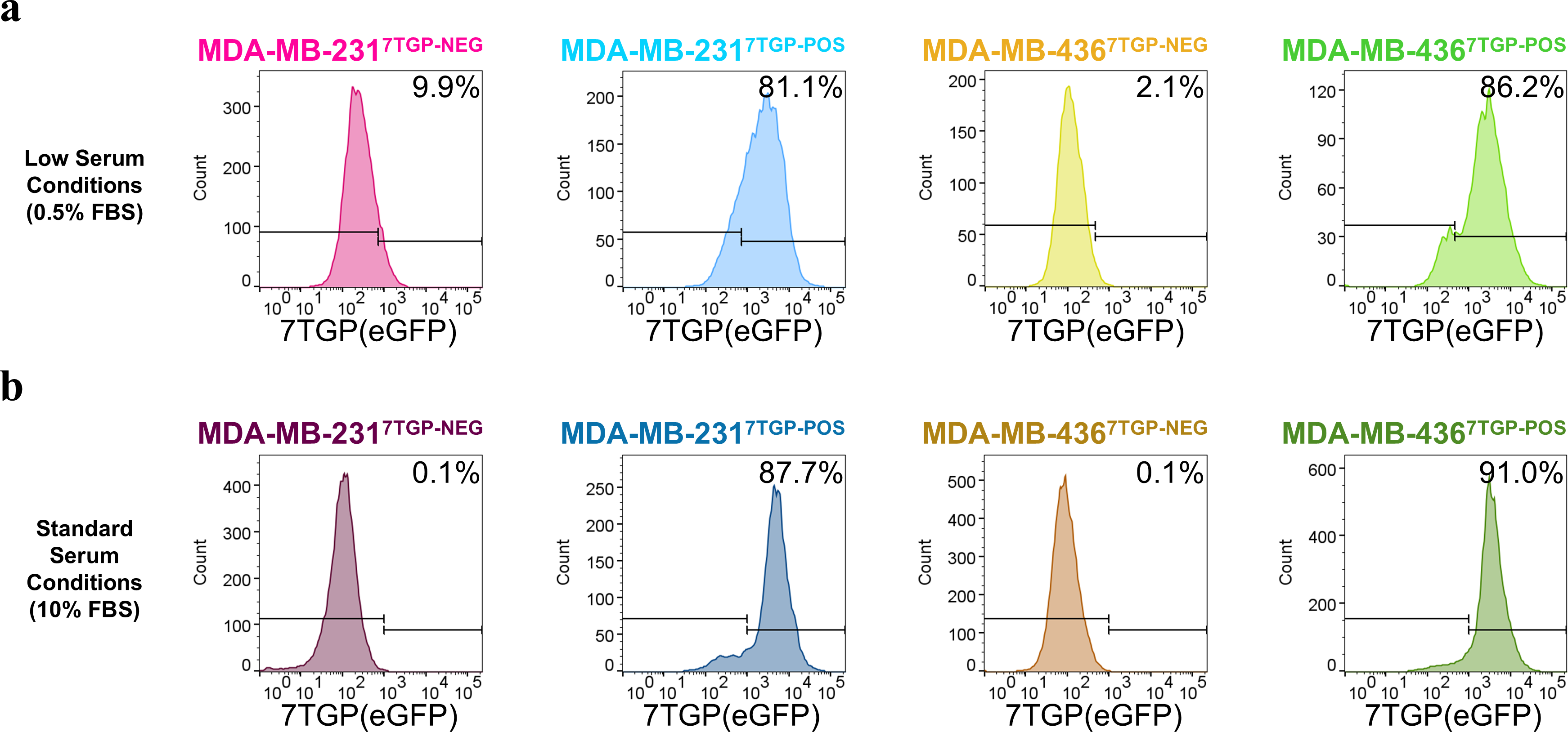


**Supplementary Fig. 1 | eGFP fluorescence profiles of Wnt-negative and Wnt-positive TNBC cell lines cultured in low or standard serum conditions. a, b** Representative flow cytometry histograms showing eGFP fluorescence in MDA-MB-231^7TGP-NEG^, MDA-MB-231^7TGP-POS^, MDA-MB-436^7TGP-NEG^, and MDA-MB-436^7TGP-POS^ cell lines after culture for 3 days in low serum (a) or 6 days in standard serum (b).

**
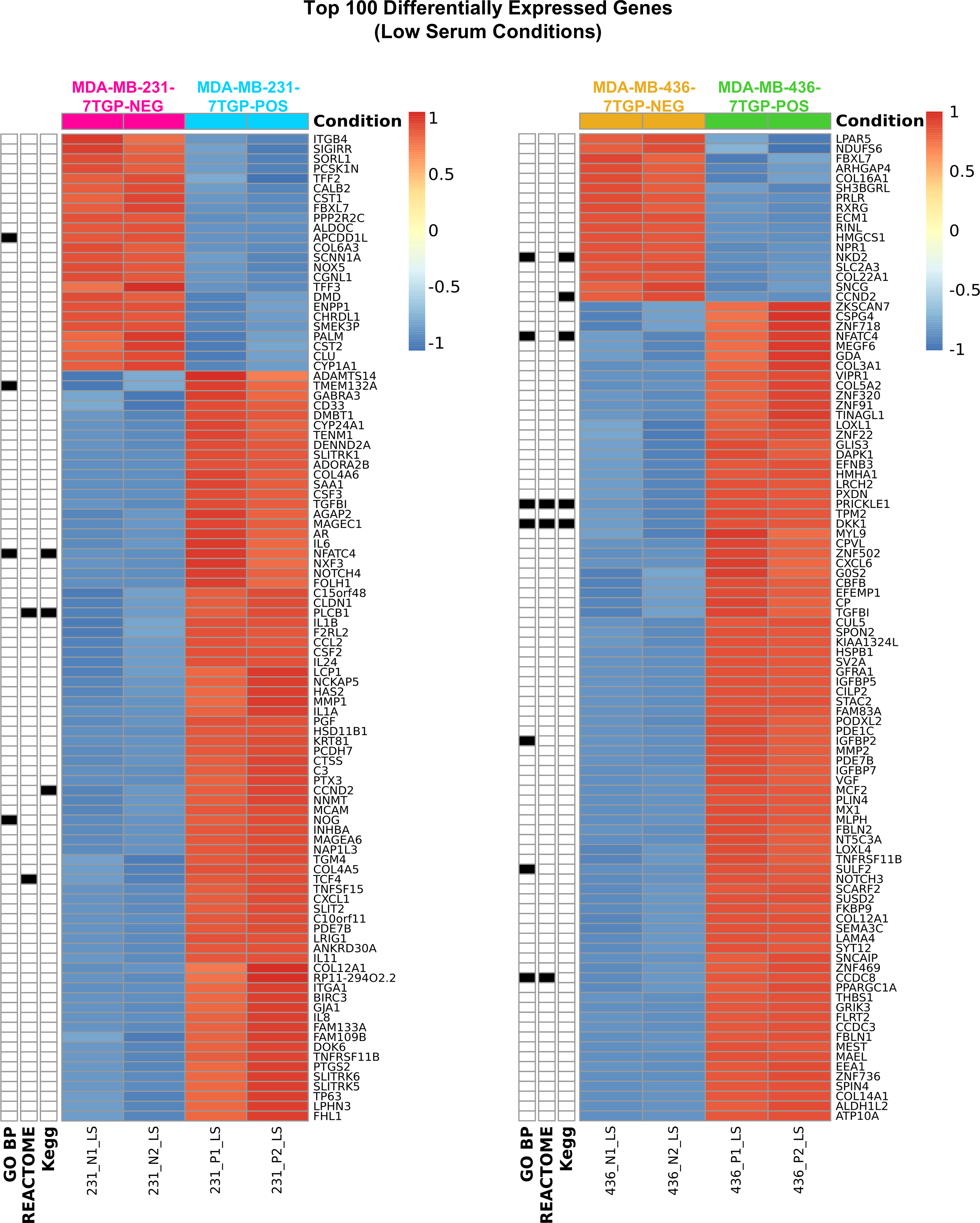
**

**Supplementary Fig. 2 | Differential mRNA expression between Wnt-positive and Wnt-negative TNBC cell lines cultured under low serum conditions.** Heat maps generated from RNA-Seq data showing the top 100 DEGs between MDA-MB-231^7TGP-POS^ and MDA-MB-231^7TGP-NEG^ cells (left panel) and between MDA-MB-436^7TGP-POS^ and MDA-MB-436^7TGP-NEG^ cells (right panel), following culture for 3 days in low serum conditions (0.5% FBS, LS). Genes previously annotated as Wnt-related, based on three gene sets (GO Biological Process: cell-cell signaling by Wnt; Reactome: signaling by Wnt; KEGG: Wnt signaling pathway), are marked in black along the y-axis. Each column represents a sample: MDA-MB-231 (231) or MDA-MB-436 (436); Wnt-negative (N) or Wnt-positive (P); replicate number (1, 2).


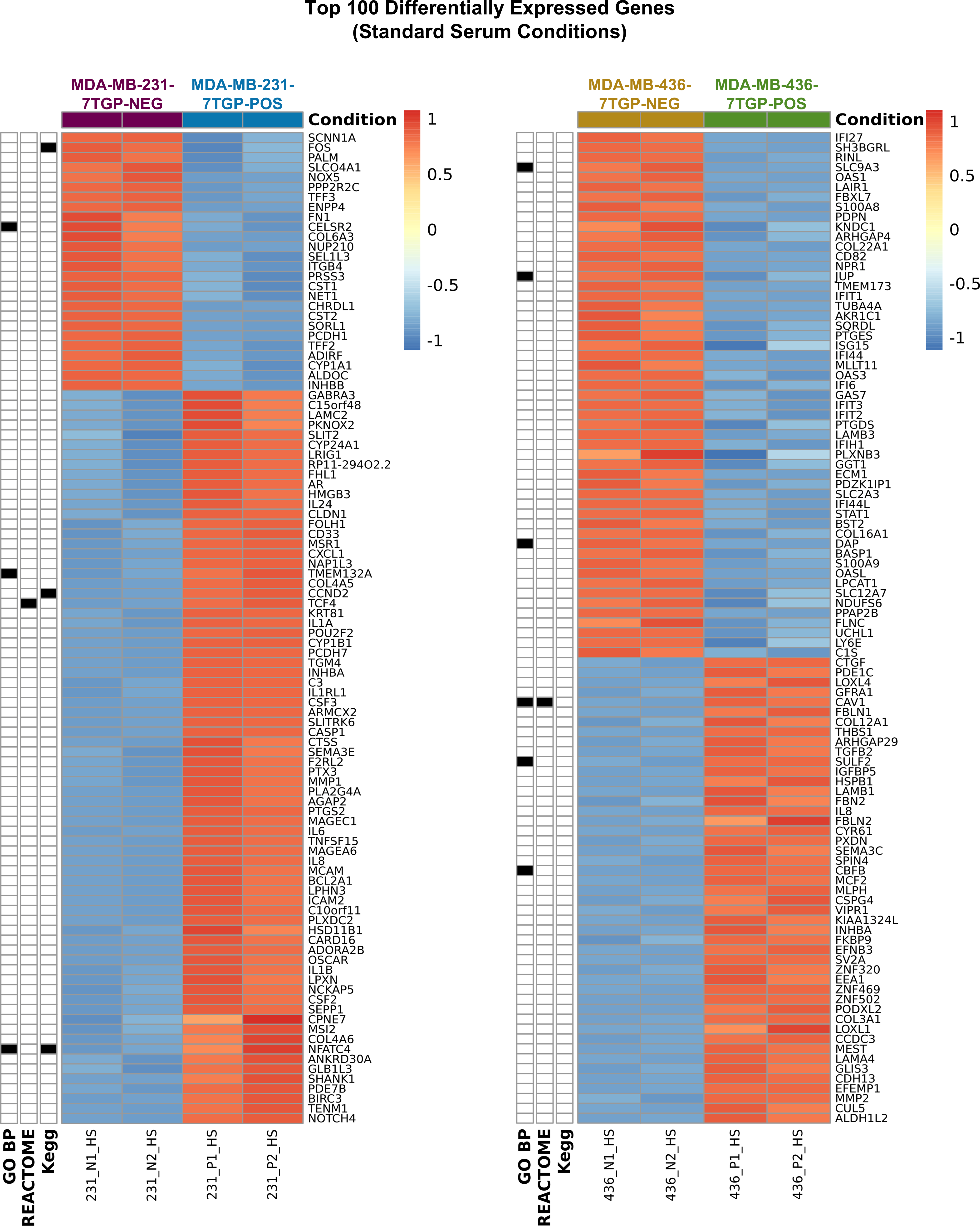


**Supplementary Fig. 3 | Differential mRNA expression between Wnt-positive and Wnt-negative TNBC cell lines cultured under standard serum conditions.** Heat maps generated from RNA-Seq data showing the top 100 DEGs between MDA-MB-231^7TGP-POS^ and MDA-MB-231^7TGP-NEG^ cells (left panel) and between MDA-MB-436^7TGP-POS^ and MDA-MB-436^7TGP-NEG^ cells (right panel), following culture for 3 days in standard serum conditions (10% FBS, HS). Genes previously annotated as Wnt-related, based on three gene sets (GO Biological Process: cell–cell signaling by Wnt; Reactome: signaling by Wnt; KEGG: Wnt signaling pathway), are marked in black along the y-axis. Each column represents a sample: MDA-MB-231 (231) or MDA-MB-436 (436); Wnt-negative (N) or Wnt-positive (P); replicate number (1, 2).


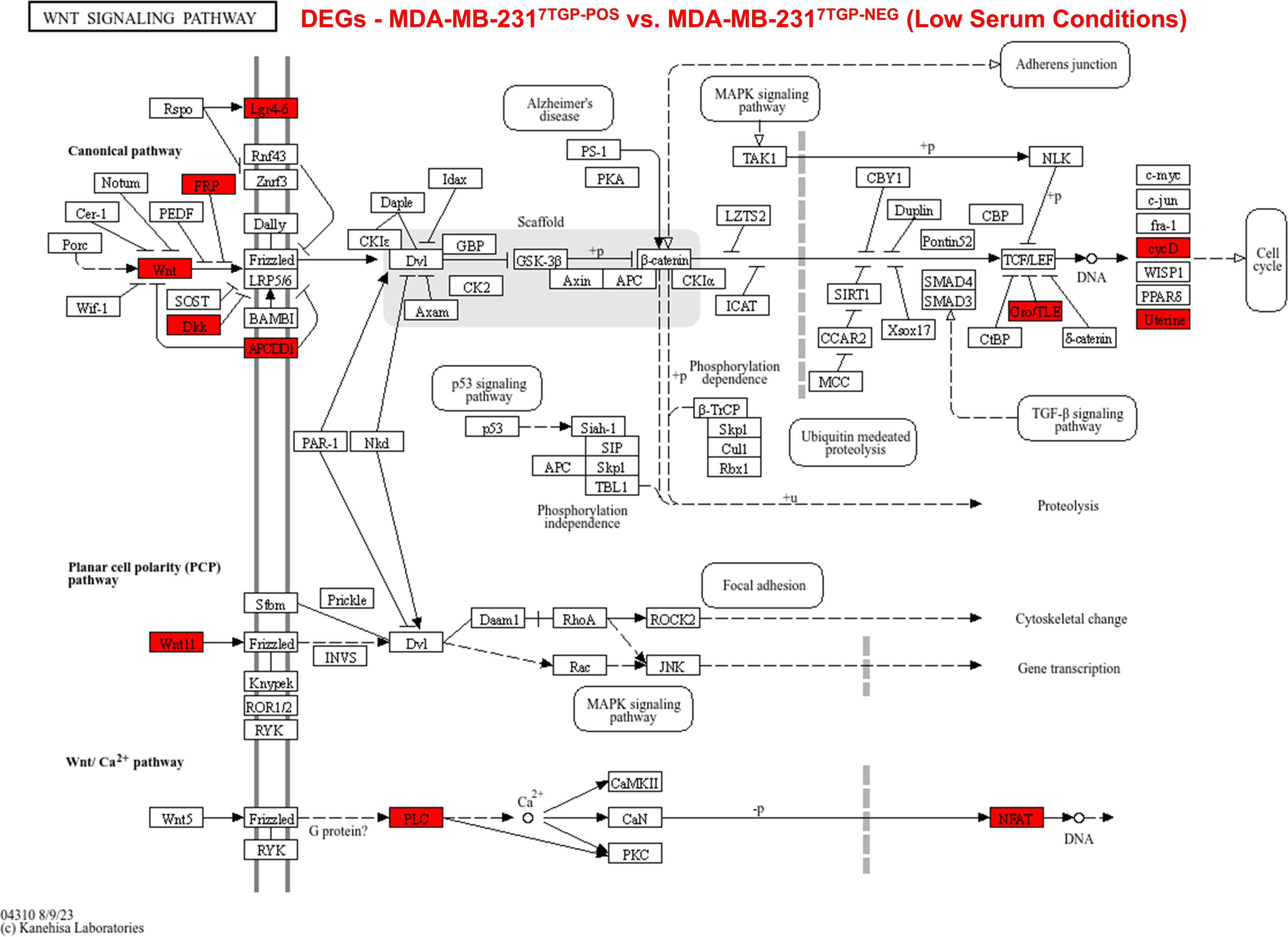


**Supplementary Fig. 4 | KEGG pathway map of Wnt signaling enriched by DEGs between MDA-MB-231^7TGP-POS^ and MDA-MB-231^7TGP-NEG^ cells.** The KEGG Wnt signaling pathway diagram (KEGG: hsa04310) highlights in red the genes or gene clusters differentially expressed between MDA-MB-231^7TGP-POS^ and MDA-MB-231^7TGP-NEG^ cells cultured in low serum. Pathway mapping was performed using KEGG Mapper (www.kegg.jp/kegg/mapper.html).


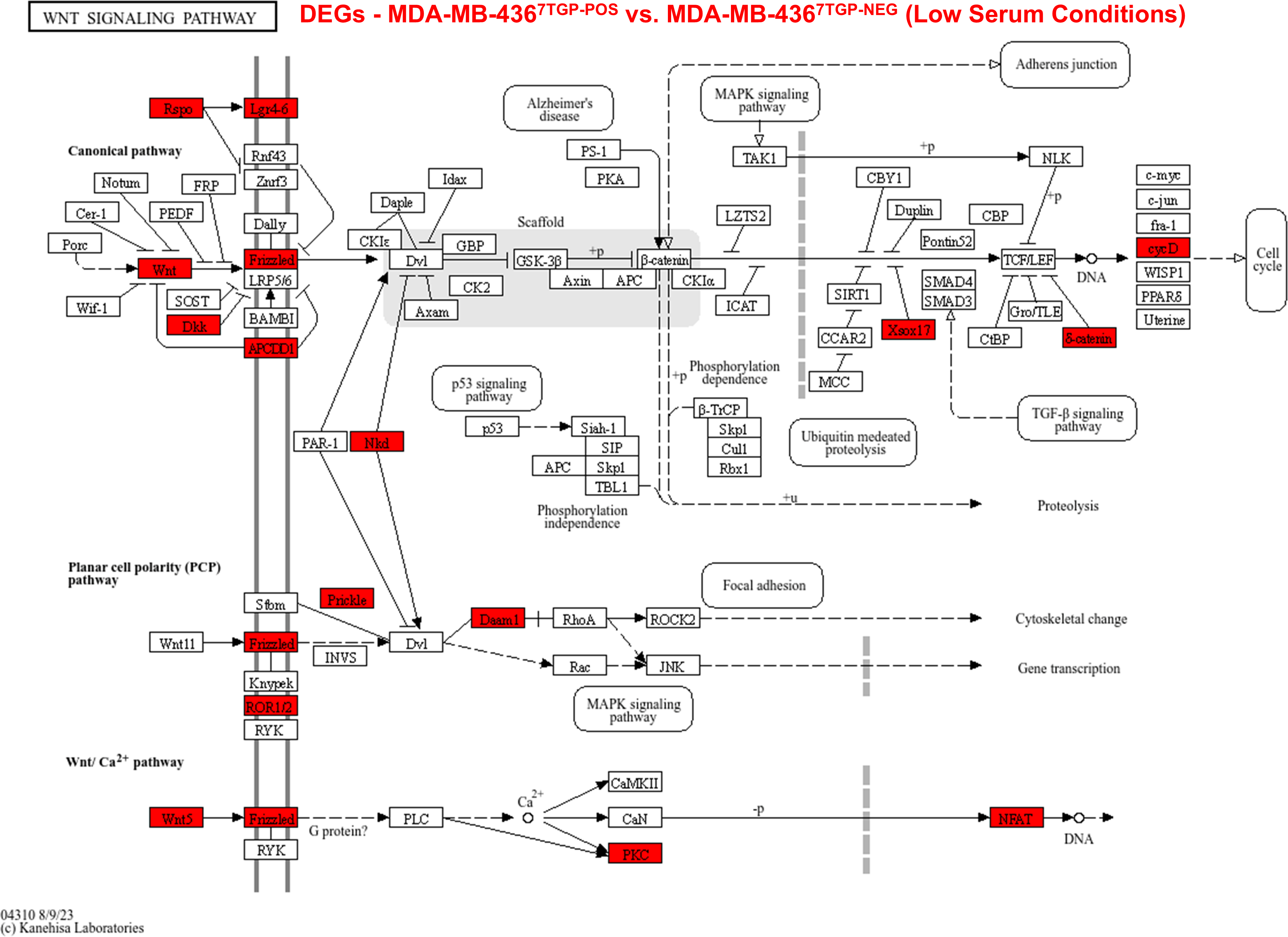


**Supplementary Fig. 5 | KEGG pathway map of Wnt signaling enriched by DEGs between MDA-MB-436^7TGP-POS^ and MDA-MB-436^7TGP-NEG^ cells.** The KEGG Wnt signaling pathway diagram (KEGG: hsa04310) highlights in red the genes or gene clusters differentially expressed between MDA-MB-436^7TGP-NEG^ and MDA-MB-436^7TGP-POS^ cells cultured in low serum. Pathway mapping was performed using KEGG Mapper (www.kegg.jp/kegg/mapper.html).


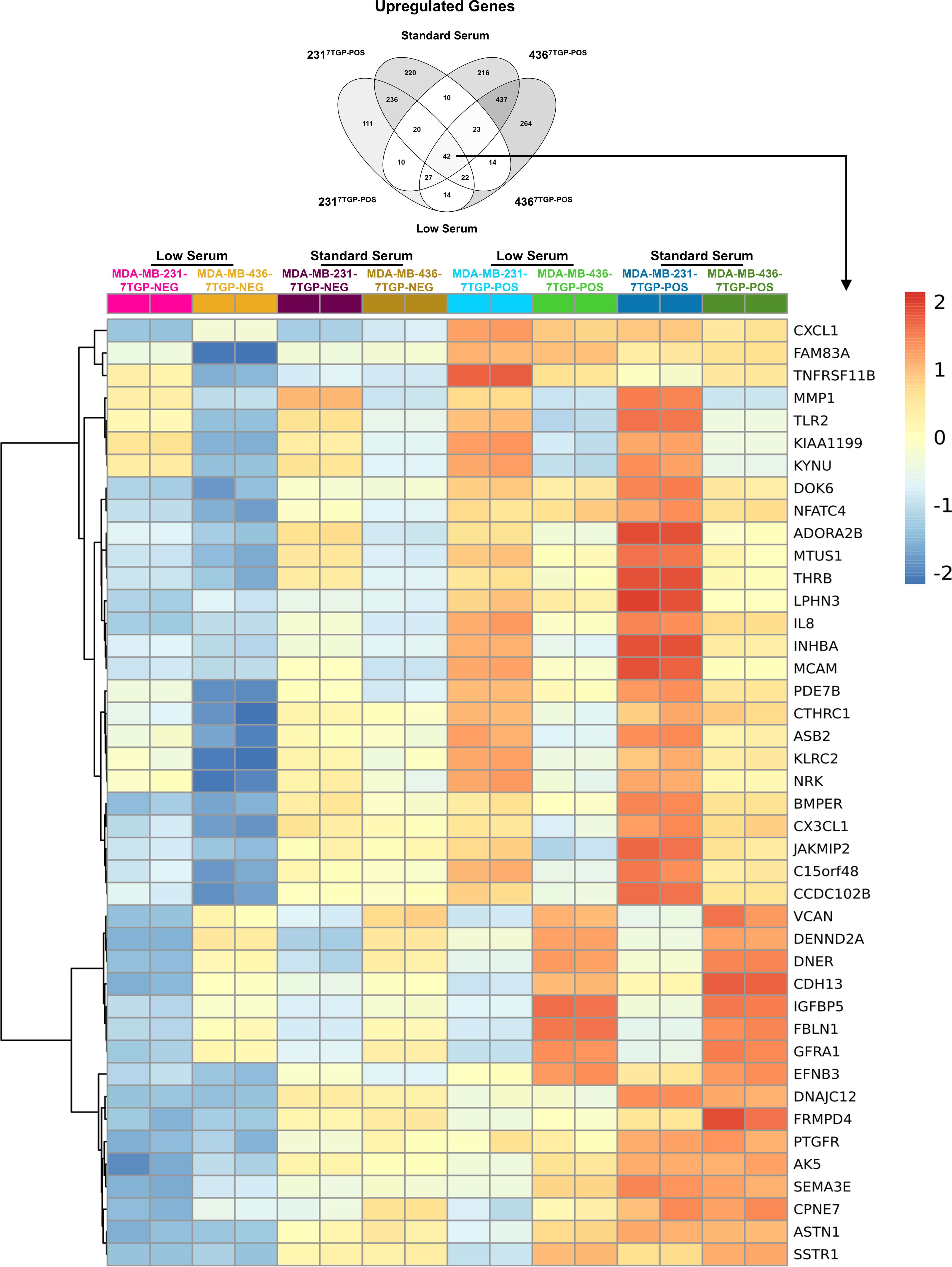


**Supplementary Fig. 6 | Shared upregulated genes across Wnt-positive TNBC cell lines and serum conditions.** Venn diagram showing the overlap of genes upregulated in Wnt-positive compared to Wnt-negative TNBC cells under both low and standard serum conditions. The heat map displays the expression patterns of the 42 genes consistently upregulated across all four comparisons. Differential expression was defined using an absolute log₂ fold change ≥ 1


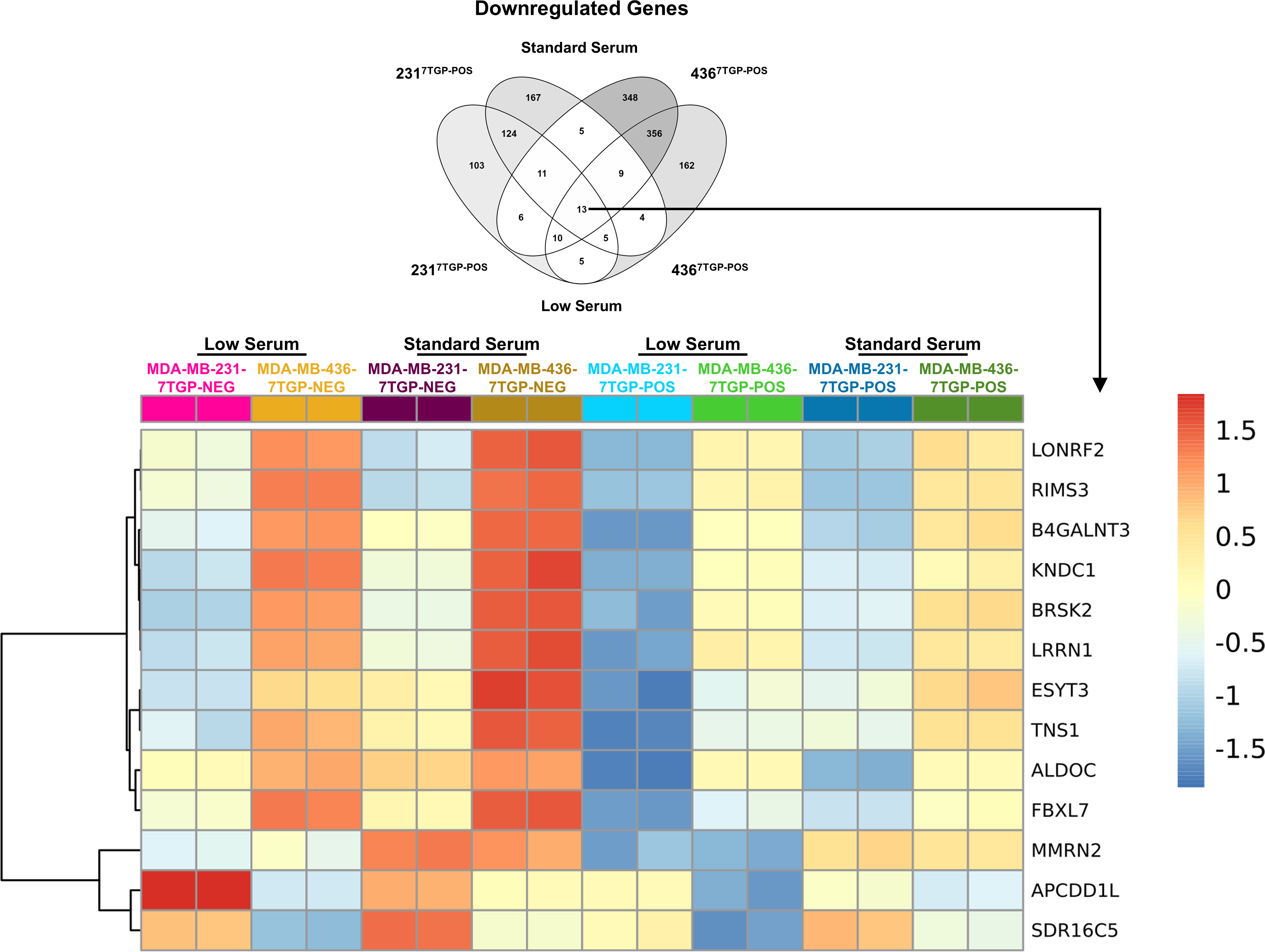


**Supplementary Fig. 7 | Shared downregulated genes across Wnt-positive TNBC cell lines and serum conditions.** Venn diagram showing the overlap of genes downregulated in Wnt-positive compared to Wnt-negative TNBC cells under both low and standard serum conditions. The heat map displays the expression patterns of the 13 genes consistently downregulated across all four comparisons. Differential expression was defined using an absolute log₂ fold change ≥ 1


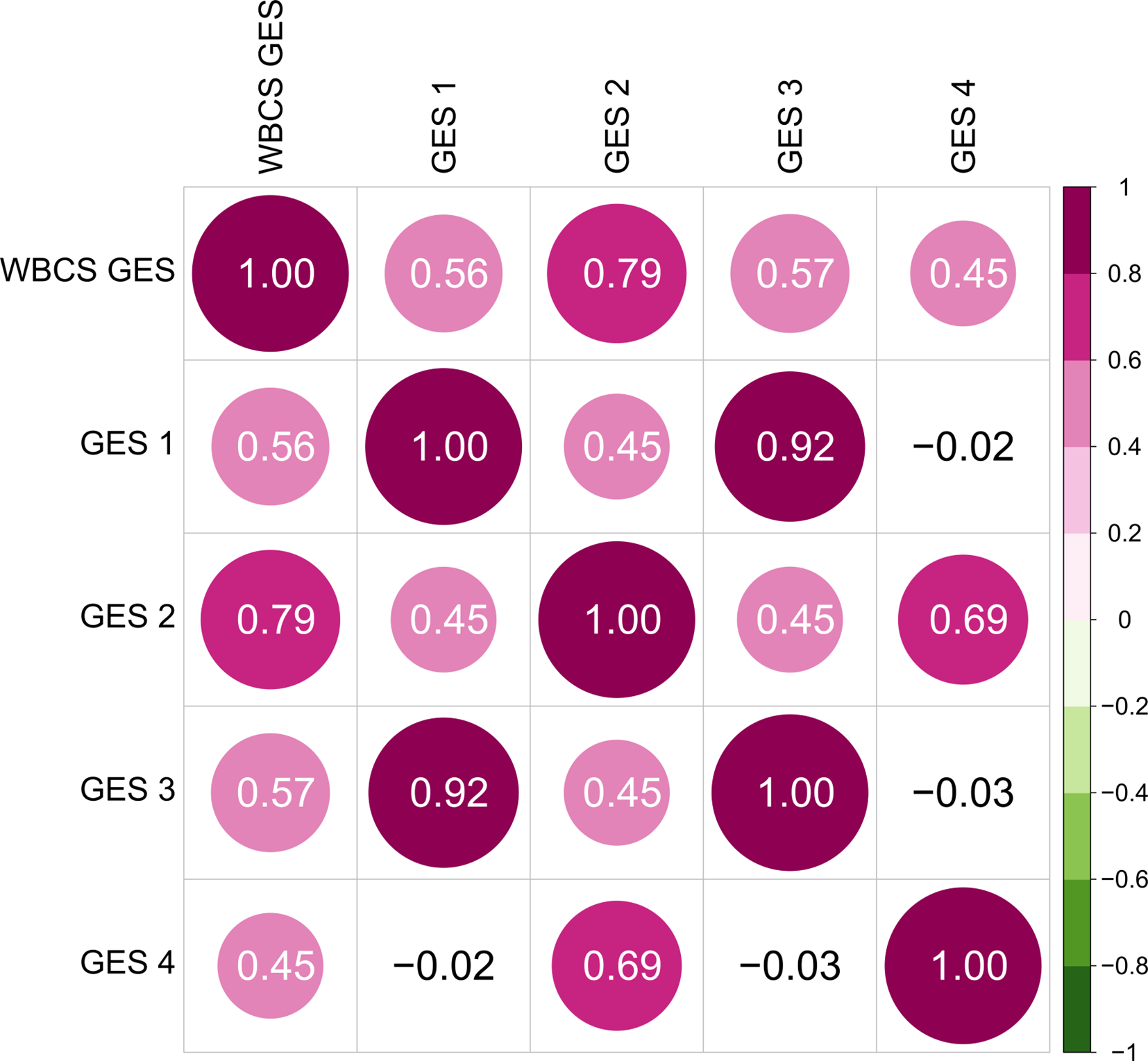


**Supplementary Fig. 8 | Correlation of Wnt-related gene expression signatures.** Spearman’s correlation analysis was performed to assess the relationship between the Wnt/β-catenin signature (WBCS) and four Wnt-related gene expression signatures (GES1-GES4). The matrix displays Pearson correlation coefficients, indicating the strength of association between signatures. WBCS: Wnt β-catenin signature from Lehmann *et al*.; GES1: 687 DEGs between MDA-MB-231^7TGP-POS^ and MDA-MB-231^7TGP-NEG^ cells cultured in low serum; GES2: 1,278 DEGs between MDA-MB-436^7TGP-POS^ and MDA-MB-436^7TGP-NEG^ cells cultured in low serum; GES3: 848 DEGs between MDA-MB-231^7TGP-POS^ and MDA-MB-231^7TGP-NEG^ cells cultured in standard serum; GES4: 1,418 DEGs between MDA-MB-436^7TGP-POS^ and MDA-MB-436^7TGP-NEG^ cells cultured in standard serum.


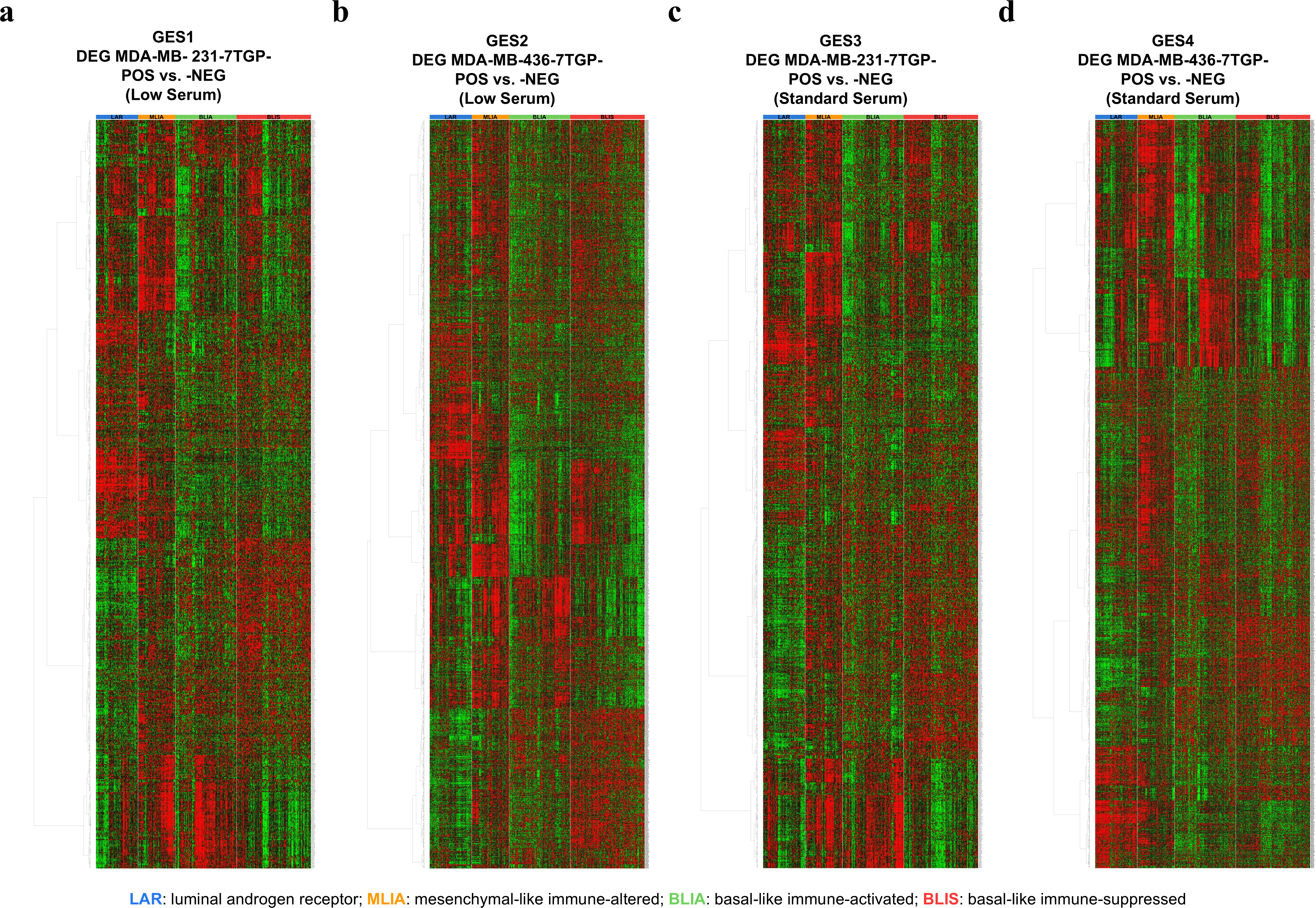


**Supplementary Fig. 9 | Expression of Wnt-related gene signatures across TNBC molecular subtypes.** Heat maps showing the expression of four Wnt-related gene expression signatures (GES1–GES4), derived from comparisons between Wnt-positive (POS) and Wnt-negative (NEG) TNBC cell lines, in a TNBC RNA-Seq cohort (n = 699). Samples are categorized into four molecular subtypes: LAR (n = 137), MLIA (n = 121), BLIA (n = 199), and BLIS (n = 242). **a**, GES1: 687 DEGs from MDA-MB-231^7TGP-POS^ vs. MDA-MB-231^7TGP-NEG^ under low serum. **b**, GES2: 1,278 DEGs from MDA-MB-436^7TGP-POS^ vs. MDA-MB-436^7TGP-NEG^ under low serum. **c**, GES3: 848 DEGs from MDA-MB-231^7TGP-POS^ vs. MDA-MB-231^7TGP-NEG^ under standard serum. **d**, GES4: 1,418 DEGs from MDA-MB-436^7TGP-POS^ vs. MDA-MB-436^7TGP-NEG^ under standard serum. Gene expression was clustered using hierarchical clustering with centered Pearson’s correlation and average linkage.


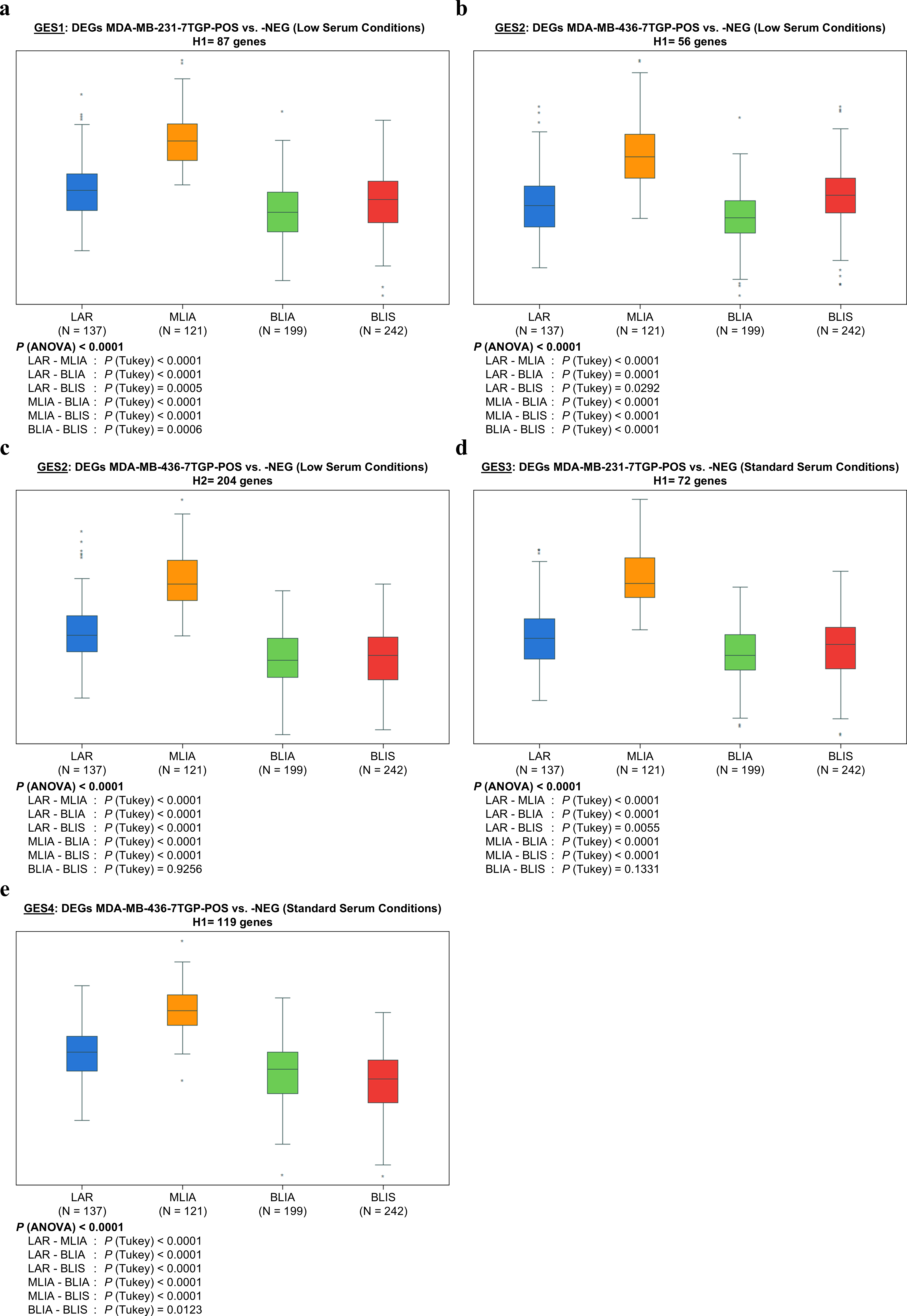


**Supplementary Fig. 10 | H1 and H2 clusters from Wnt-related gene signatures are differentially enriched across TNBC subtypes.** H1 and H2 gene clusters derived from Wnt-related gene expression signatures were evaluated in a TNBC RNA-Seq cohort (n = 699) comprising four molecular subtypes: luminal androgen receptor (LAR), mesenchymal-like immune-altered (MLIA), basal-like immune-activated (BLIA), and basal-like immune-suppressed (BLIS). Clusters were identified by hierarchical clustering using centered Pearson’s correlation. Bar plots show enrichment scores for each cluster across TNBC subtypes:

**a**, H1 GES1 (87 genes), from MDA-MB-231^7TGP-POS^ vs. MDA-MB-231^7TGP-NEG^ under low serum. **b**, H1 GES2 (56 genes), and **c**, H2 GES2 (204 genes), from MDA-MB-436^7TGP-POS^ vs. MDA-MB-436^7TGP-NEG^ under low serum. **d**, H1 GES3 (72 genes), from MDA-MB-231^7TGP-POS^ vs. MDA-MB-231^7TGP-NEG^ under standard serum. **e**, H1 GES4 (119 genes), from MDA-MB-436^7TGP-POS^ vs. MDA-MB-436^7TGP-NEG^ under standard serum. Statistical significance was assessed using one-way ANOVA with Tukey’s post hoc test. *P* values ≤ 0.01 were considered significant and are shown below each panel.


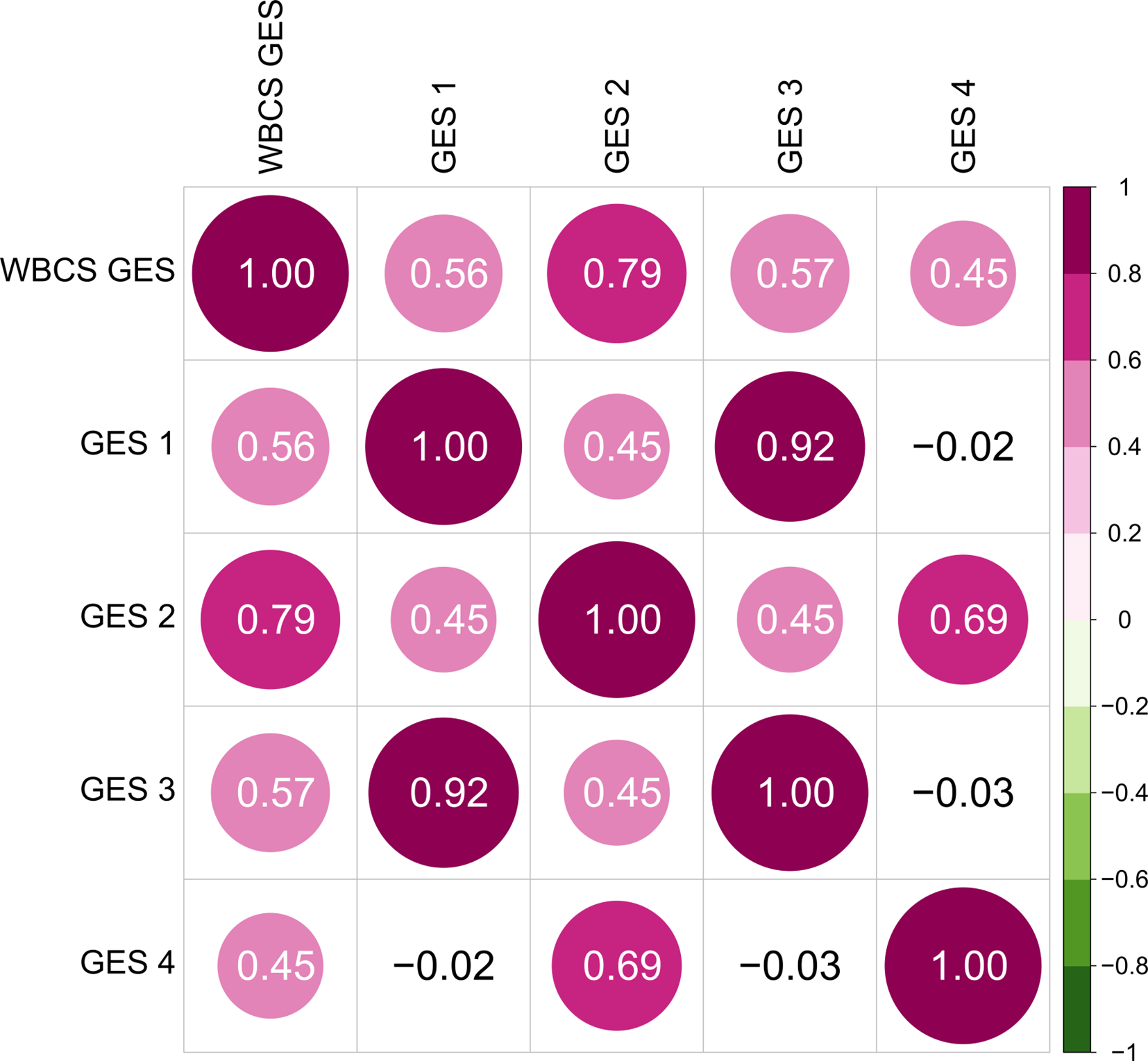


**Supplementary Fig. 11 | Correlation of H1 and H2 clusters from Wnt-driven gene signatures with the Wnt/β-catenin signature.** Spearman’s correlation analysis was used to assess the relationship between the Wnt/β-catenin signature (WBCS) from Lehmann *et al*. and five Wnt-related H1 and H2 gene clusters derived from hierarchical clustering of gene expression in a TNBC RNA-Seq cohort (n = 699). The matrix displays Pearson correlation coefficients, reflecting the degree of association between each cluster and the WBCS. H1 GES1: 87 DEGs between MDA-MB-231^7TGP-POS^ and MDA-MB-231^7TGP-NEG^ cells cultured in low serum; H1 GES2: 56 DEGs between MDA-MB-436^7TGP-POS^ and MDA-MB-436^7TGP-NEG^ cells cultured in low serum; H2 GES2: 204 DEGs between MDA-MB-436^7TGP-POS^ and MDA-MB-436^7TGP-NEG^ cells cultured in low serum; H1 GES3: 72 DEGs between MDA-MB-231^7TGP-POS^ and MDA-MB-231^7TGP-NEG^ cells cultured in standard serum; H1 GES4: 119 DEGs between MDA-MB-231^7TGP-POS^ and MDA-MB-231^7TGP-NEG^ cells cultured in standard serum.
